# Facile Synthesis of Mayenite Electride Nanoparticles Encapsulated in Graphitic Shells Like Carbon Nano Onions: Non-noble-metal Electrocatalysts for Oxygen Reduction Reaction (ORR)

**DOI:** 10.3389/fchem.2019.00934

**Published:** 2020-01-22

**Authors:** Karim Khan, Ayesha Khan Tareen, Muhammad Aslam, Yupeng Zhang, Renheng Wang, Sayed Ali Khan, Qudrat Ullah Khan, Muhammad Rauf, Han Zhang, Zhengbiao Ouyang, Zhongyi Guo

**Affiliations:** ^1^School of Electrical Engineering & Intelligentization, Dongguan University of Technology (DGUT), Dongguan, China; ^2^College of Electronic Science and Technology, THz Technical Research Center of Shenzhen University, Key Laboratory of Optoelectronics Devices and Systems of Ministry of Education and Guangdong Province Shenzhen University, Shenzhen, China; ^3^Shenzhen Engineering Laboratory of Phosphorene and Optoelectronics, SZU-NUS Collaborative Innovation Center for Optoelectronic Science and Technology, Shenzhen University, Shenzhen, China; ^4^Government Degree College PaharPur, Gomel University, Dera Ismail Khan, Pakistan; ^5^Shenzhen Key Laboratory of Flexible Memory Materials and Device, College of Electronic Science and Technology, Shenzhen University, Shenzhen, China; ^6^College of Chemistry and Environmental Engineering, Shenzhen University, Shenzhen, China

**Keywords:** synthesis of GS-CNOs, mayenite electride, new electrocatalyst, ORR, fuel cells

## Abstract

This manuscript presented a large scale synthesis of Graphitic Shells like carbon nano onions (GS-CNOs) by direct solution method using mayenite electride as a catalyst for synthesis of CNOs. Thermal characterization, microstructural analysis, and high resolution electron microscopy have confirmed the graphitization and revealed the resulting GS-CNOs with particle size about 15 nm, maximum BET surface area of 214 m^2^.g^−1^, and moderate conductivity of 250 S.cm^−1^, thus providing a new approach to synthesize GS-CNOs. The reported GS-CNOs, which acts as more active but less expensive electrocatalysts with onset potential of 1.03 V, half wave potential of 0.88 V vs. the reversible hydrogen electrode (RHE), and limited current density of 5.9 mA.cm^−2^, higher than that of benchmark 20% Pt/C (1.02 eV, 0.82 V, 5.2 mA.cm^−2^). The synthesized nano-powder acts as an origin of ORR activity via a four electron (4e^−^) pathway, along with significantly enhanced stability, in alkaline media. The high ORR activity is ascribed to GS-CNOs embedded sufficient metallic C12A7:e^−^ particles, which favor faster electron movement and better adsorption of oxygen molecules on catalyst surface. Hence, we explored first time large scale synthesis of GS-CNOs with gram level and provide efficient approach to prepare novel, lowest cost, potential non-noble metals catalyst for fuel cells.

**Graphical Abstract:**
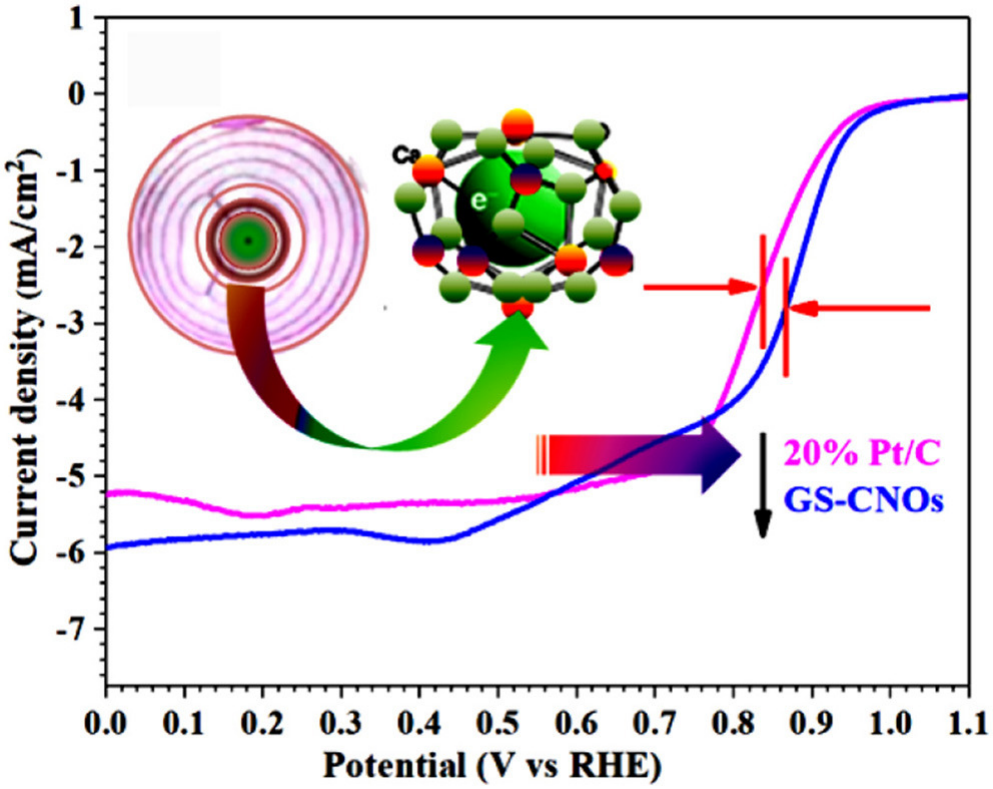
A large scale synthesis of Graphitic Shells like carbon nano onions (GS-CNOs) are synthesized by direct solution method using mayenite electride as a catalyst for synthesis of CNOs and studied their ORR properties.

## Introduction

Technologies retrospect and the advancements in the already existing technology; usually come with the development of new materials (Tahir et al., 2015; Huang et al., 2016; Jiang et al., 2016; Khan et al., 2016, 2018a,b,c,d,e, 2019a,b,c, 2020; Wang et al., 2016; Zhang et al., 2016; Ren et al., 2017; Zou et al., 2017; Li et al., 2018; Mahmood et al., 2018). Fuel cells technology is considered as a key component in energy related applications with pollution free environment. The proton exchange membrane fuel cells (PEMFC) heavily depend on Pt-based electrocatalysts (Bai et al., 2015; Huang et al., 2016; Zhang et al., 2016), which have prohibited their widespread utilization (Jian et al., 2017, 2019; Tabassum et al., 2017; Han et al., 2018a,b, 2019a,b; Khan et al., 2018e, 2019a,b; Mahmood et al., 2019; Tareen et al., 2019a). To overcome this obstacle, the alkaline anion exchange membrane fuel cells (AAEMFC) were proposed. By switching from acidic to alkaline based electrolyte, it is possible to use non-noble metal based electrocatalysts materials as a cathode, because in acidic medium it needs large quantity of Pt based materials. However, the sluggish ORR kinetics at cathode is taken as a main cause for the low efficiency of next-generation electrocatalysts based energy-conversion devices. The ORR activity as well as durability has attained increasing attention for potential substitution of Pt by non-noble metals based catalysts.

Conventional precious metals based electrocatalysts have some limitations in utilization due to high cost (Tareen et al., 2019b). The innovation and progress of electrode materials assure higher energy and power density (Sun et al., 2018). Hence, economical, highly stable, good-performance catalysts synthesis that is free of platinum group metal (PGM) for sluggish ORR is important to industrialize large-scale in fuel cells (Chatterjee et al., 2018). For this reason, carbon family is among one of the most attractive materials system in academic research and industrial point of view due to its incredible diversity and flexibility with low cost, abundant nature, excellent properties and potential industrial applications. Their properties fluctuate with respect to their allotropic form and also significantly rely on their morphology, structure, and surface composition. Carbon black as well as activated carbon are most conventional one, but with various nano-structured forms like graphene, fibers, nano-tubes, and mesoporous morphologies are emerging recently (Choi and Kim, 2017). The story of carbon family started with fullerenes, moving to carbon nanotubes, finally to graphene, are widely used in many potential industrial applications including electronics, tribology, energy producing and storage (Khan et al., 2018c).

Among other carbon family members, we are interested in CNOs. CNOs are multi-layer fullerenes, discovered by Ugarte (1992) which are concentric shells of carbon atoms (Bartelmess and Giordani, 2014). CNOs comprise a new class of carbon allotropes, with high surface area (30–500 m^2^/g), good electric conductivity, excellent thermal stability, and appropriate mesoporous microstructure (Borgohain et al., 2014). CNOs are important materials which are applicable in many fields. Despite the level of effort on the synthesis and applications of pristine and composite form of CNOs, their potential uses in energy conventional have not yet been well-recognized. Therefore, production of pure, large scale CNOs with lowest cost is very excited. Over the last few years, a variety of methods have been demonstrated for synthesis of CNOs, e.g., electron irradiation (Ugarte, 1992), ion implantation (Banhart et al., 1997), counter-flow diffusion flames (Hou et al., 2009), CVD using transition metal catalysts (Fei et al., 2006), plasma-enhanced CVD (Chen et al., 2001), annealing nanodiamonds (Kuznetsov et al., 1994), plasma spraying of the nanodiamonds (Gubarevich et al., 2003), and underwater arc discharge (Sano et al., 2002). According to best of our knowledge, all these methods are with low or moderate yield and require subsequent separation steps to get material with acceptable purity. Therefore, a quantitative synthesis of CNOs is required with low-cost, scalable and high quality pure CNOs for downstream applications. CNOs diameter depends on synthetic protocol, nevertheless, CNOs exhibit in wide-range high surface area to volume ratio. The CNOs had a lot of the applications as a biological imaging/sensing, capacitors, catalysis, tribology, environmental remediation (Choi and Kim, 2017), lithium-ion batteries (LIBs), terahertz-shielding, optical limiting, molecular junctions in STM, fuel cell, etc. (Khan et al., 2018e). The CNOs has desirable an electrical conductivity and surface area, higher than CNTs make them potential electrocatalytic ORR applicants for advancement of fuel cells (Xu et al., 2006).

Regarding energy applications everyone is interested in the green energy for protecting environment from air pollution. The ORR in the fuel cell technology is important reaction and its performance determined by a lot of factors (Khan et al., 2018e). Commercially used benchmark Pt-based catalysts drawbacks are its high price, low stability under some applied conditions, limited its applications in fuel cell. So, high-performance PGM- and Fe-free catalysts are highly desirable for fuel cell technology. Hence, inexpensive, highly active, and more stable catalyst for example nitrides, alloys, doped carbon based materials, and their complexes with metal are introduced but with some limitations (Khan et al., 2018e). Recent progress in CNOs (Choi and Kim, 2017) as efficient cathode electrocatalysts for ORR in fuel cell has been done with limitations synthesis method. Based on our previous knowledge and experience through our previous work on mayenite electride, [Ca_24_Al_28_O_64_]^4+^. (4e^−^) (thereafter, C12A7:e^−^, where C = CaO, and A = Al_2_O_3_) catalysts provides guidelines for synthesis of GS-CNOs embedded C12A7:e^−^ catalysts with good stability, and more active sites with mesoporous structure. Recently, motivated unique opportunities offered by C12A7:e^−^ catalyst, there is a lot of interest in this direction, encouraged by the rising production methods and applications. Therefore, to further get a purified GS-CNOs with low cost, we tried to use C12A7:e^−^ as catalyst for synthesis of GS-CNOs, because C12A7:e^−^ is also very active and stable electrocatalysts for the ORR (Khan et al., 2018e). The important theoretical study based on DFT calculations predicted that metallic particles moieties embedded in the carbon have comparable activity and enhanced stability (Li et al., 2018). Inspired by this theoretical prediction, we hypothesized that C12A7:e^−^ embedded in GS-CNOs will probably more stable and also solve the grand challenge of increasing density of the active sites in catalysts because C12A7:e^−^ is more stable and active metallic compounds with low work-function (Khan et al., 2018b,c,e).

## Synthesis of GS-CNOs

**Figure 1:**
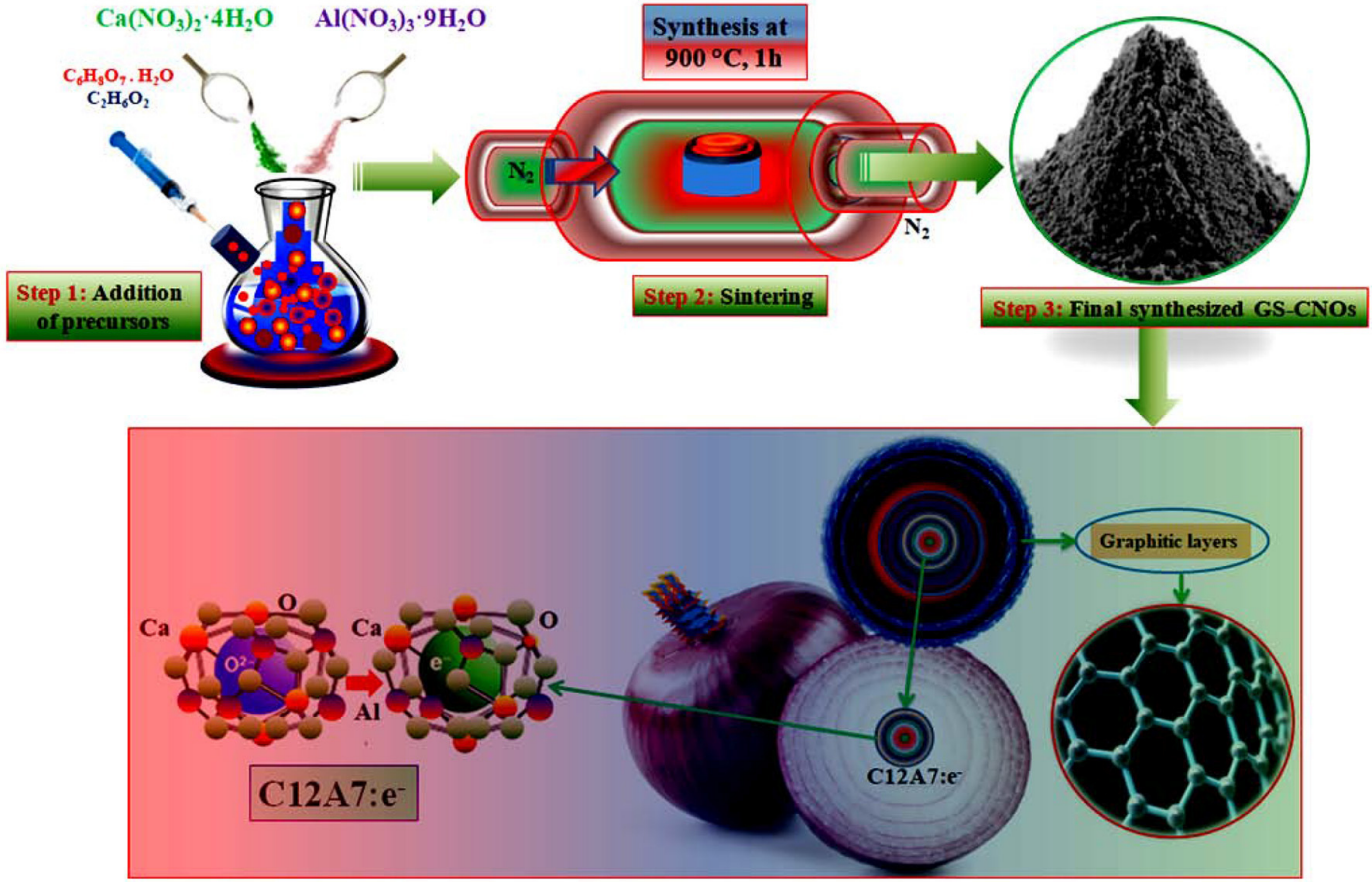
Schematic representation of GS-CNOs composite synthesis.

Selecting appropriate synthesis conditions is very important because selected applied conditions strongly affect the properties of final material. A continuous single-step synthesis scheme of the mesoporous GS-CNOs embedded C12A7:e^−^ is shown in Figure 1, will provide gram level synthesis of comparatively pure and high surface area GS-CNOs. The schemed citrate sol-gel method was applied for synthesis of required materials at particular applied temperature, with and without C12A7:e^−^ catalyst, under same applied condition. Initially, we mixed citric acid (CA) in ethylene glycol (EG) at 75°C in 4:1 ratios. To obtain transparent solution, the precursors solution was heated it at 90°C along with continuous stirred. This gel was kept at about 120°C for 1 h to vaporize physically absorbed water and then finally dried at 290°C for 2 h in a drier. The resulting dried gel was further heat treated at 550°C for 90 min in nitrogen (N_2_) environment with 5°C/min increase/decreasing rate, followed by final sintering under N_2_ atmosphere at 900°C for 1 h, with a heating rate of 4°C/min. This synthesized material was like an Aero-gel, so we called in further manuscript it as Aero-gel material.

On other hand, for electrocatalytic applications, we also synthesized nanosize, metallic particles moieties embedded in the carbon. So, to get high surface area nanosized embedded particles, we repeatedly applied the same synthesis conditions, as we mentioned above, with extra added of Ca and Al nitrates (12:14, to get 12CaO.7Al_2_O_3_ (also called as mayenite electride, with short cut name of C12A7:e^−^) in CA and EG (4:1), to get mayenite electride, C12A7:e^−^ nano-particles embedded in carbon. In details, first of all metals nitrates, Ca(NO_3_)_2_·4H_2_O and Al(NO_3_)_3_·9H_2_O were weighted in ratios of 12:14 to get C12A7:e^−^ and added to the EG and CA solution (4:1) at the same time (Khan et al., 2016; Zou et al., 2017). In addition, we applied the same heat treatment conditions, as we mentioned above and finally obtained GS-CNOs under N_2_ atmosphere with a heating rate of 4°C/min and kept at temperature of 900°C for 1 h. The synthesized materials was then used for an initial investigation by using different experimental methods based on synthesis ways from precursors to final conductive powder.

Here different characterizations were carried out via different techniques, like X-ray powder diffraction (XRD) analysis was performed to investigate the obtained phase(s) crystallinity. Brunauer-Emmett-Teller (BET) was used to measure specific surface area and pores volume/width of samples. Four probe methods were used to measure electrical conductivity. Scanning electron microscope (SEM) and high resolution transmission electron microscope (HR-TEM) were used to study the microstructure and morphology of the final products.

Finally, in case of electrochemical measurements, all electrochemical measurements were performed in 0.1 m KOH solutions using a three-electrode electrochemical workstation system (CHI 760E, CH Instruments, Inc.). Electrochemical measurement was carried out first via ink preparation by dissolving GS-CNOs composite powder in mixture of water, isopropyl alcohol, and Nafion with volume ratios 1:9:0.1, and sonicated it for 30 min at room temperature. The prepared 8 μL ink was dropped onto glassy carbon electrode (GCE) (0.196 cm^−2^) and dried slowly under atmospheric conditions, to get fine coated film on GCE surface. The catalyst loading of active GS-CNOs embedded C12A7:e^−^ on working GCE was ~0.1 mg.cm^−2^. Cyclic voltammetry (CV) data was calculated after purging O_2_ for at least 20 min, at 300 K. Later, electrode was introduced to electrochemical setup at potential cycling between 0.05 and 1.2 V at 100 mV·s^−1^ in alkaline electrolyte (0.1 M KOH), until stable CV curves were obtained after 10,000 cycles. Consequently, for rotating disk electrode (RDE) measurements, background capacitive currents were recorded in a potential range from 1.2 to 0.2 V in N_2_-saturated electrolyte, with scan rate of 10 mV·s^−1^. For alkaline ORR measurements, the loading amount of 20% Pt/C was ~0.1 mg.cm^−2^, which contain loaded quantity of Pt was ~20 μg cm^−2^. Finally, the linear sweep voltammograms (LSV) in O_2_-saturated electrolyte were measured at different rotation speed from 400 to 2,500 rpm. The LSV at 1,600 rpm of GS-CNOs composite electrode was compared with commercially used benchmark 20% Pt/C. We will discuss onward each characterization based obtained results in details.

## Results and Discussions

### Phase and Microstructural Analysis

The synthesized material under same applied conditions shows the different appearance, without C12A7:e^−^ was Aero-gel type (Figure S1) and with the C12A7:e^−^ shows GS-CNOs type nanosize particles, under same applied temperature and environmental conditions. Here, in this paper we are more interested in synthesis and applications of GS-CNOs for electrocatalytic applications because of their higher BET specific surface area, durability and enhanced expected stability due to addition of the C12A7:e^−^. So, in next section we will discuss in detail the characterizations about well spherical shaped nanosized GS-CNOs and most importantly we will concentrate on their applications for ORR in fuel cell.

### Crystalographic Study

**Figure 2:**
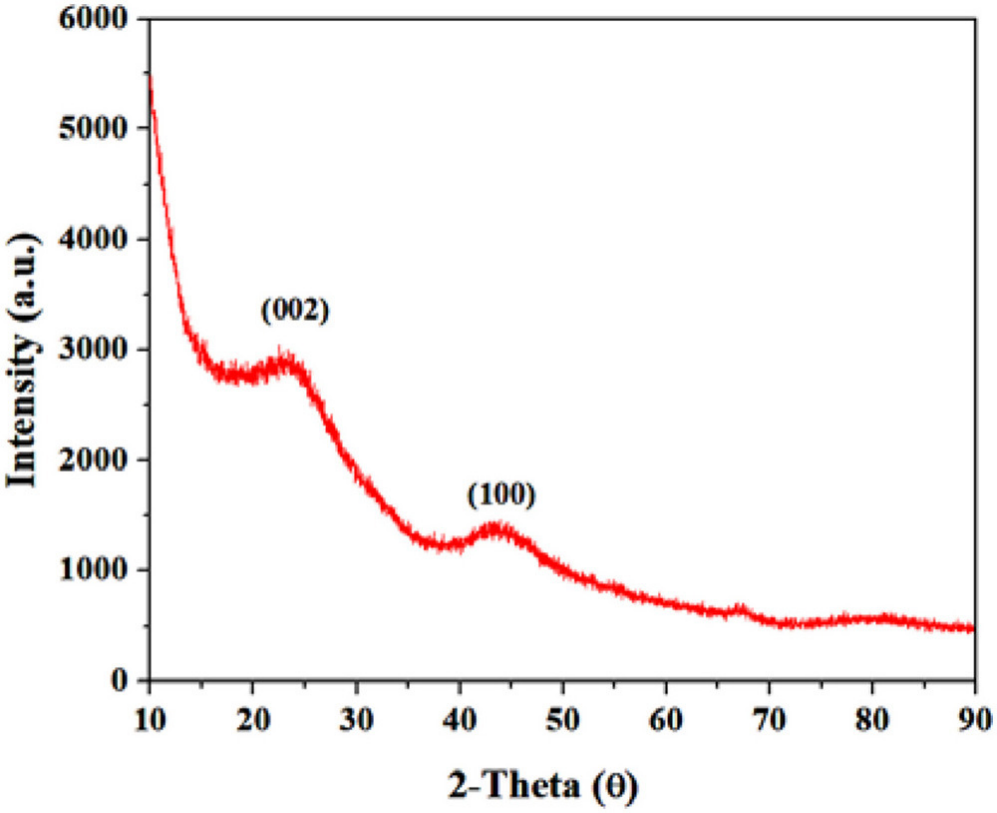
XRD of GS-CNOs synthesized at 900°C, 1 h.

First of all, XRD was used to study the crystalline phase of synthesized GS-CNOs (Figure 2). The obtained XRD of the GS-CNOs, has two peaks, one at 2θ = 25.2° and second at 2θ = 43.6° (Zhou et al., 2011). These two peaks can be assigned as typical graphitic (002) and (100) planes, respectively (Choi and Kim, 2017). To further evaluate the obtained results and study the formation of graphitized structure, we studied Raman spectroscopy of GS-CNOs.

### Raman Spectroscopy

**Figure 3:**
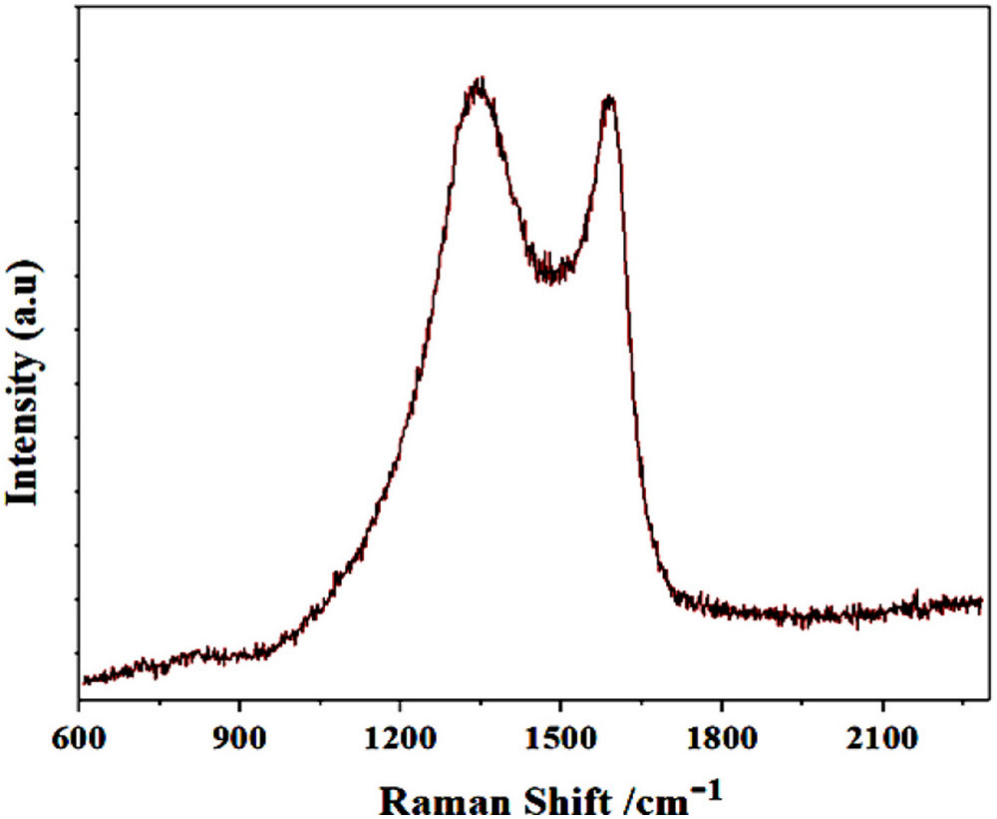
Typical Raman spectra of pristine GS-CNOs synthesized at 900°C, 1 h.

The Raman spectroscopy is among the rapid and relatively low cost characterization technique for the identification of carbon family. Typically, for carbon family there are two wide Raman bands those are laid between 1,250 and 1,600 cm^−1^. Here, in our case, the Raman spectrum of synthesized GS-CNOs, shows two stretching peaks at 1,350 and 1,580 cm^−1^, corresponding to D and G band peak, respectively (Figure 3) (Choi and Kim, 2017; Khan et al., 2018b,c,e). For further analysis of specific surface area of synthesized material, we did BET analysis, because the catalytic activity highly dependent on the specific surface area.

### Specific Surface Area of Synthesized GS-CNOs

**Figure 4:**
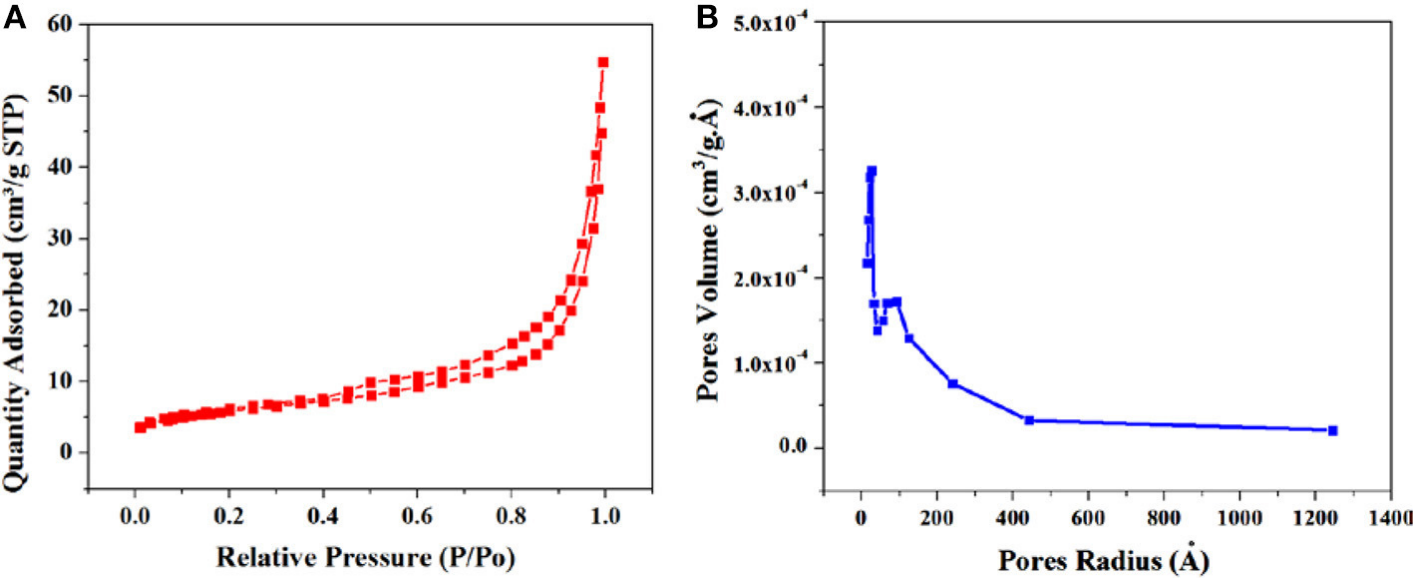
**(A)** BET results, **(B)** BJH curve of GS-CNOs composite synthesized at 900°C, 1 h.

The BET theory is based on the physical adsorption-desorption of gas molecules on solid surface of material by which specific surface area can calculated (Figure 4). The predicted BET surface area of the GS-CNOs was 214 m^2^.g^−1^. Similarly, under same applied conditions synthesized material without C12A7:e^−^ was like Aero-gel with the estimated calculated BET surface area was 11 m^2^.g^−1^ (Figure S2), lower then GS-CNOs synthesized under same conditions. This significant increase in surface area can be related to the catalyst, C12A7:e^−^. In case of GS-CNOs, the adsorption of nitrogen on GS-CNOs showed like isotherm IV type, which is typical for mesoporous materials. Similarly, the carbon aero-gel (Figure S2), carbonized at 900 °C, also exhibit a type IV isotherm. Estimated pore diameters were ~11–80 nm and ~19 nm, for without and with catalyst composite, well-agreement with Barrett-Joyner-Halenda (BJH) method. The catalyst based synthesized GS-CNOs has greater mesopore volume of 0.35 ± 0.04 cm^3^.g^−1^ compared to aero-gel (0.085 cm^3^.g^−1^). Hence, it is clear that addition of C12A7:e^−^ catalyst appears to have increased the prevalence of mesopores with variable diameters and hence can be used to affect both macroporous and mesoporous structure. As a result it will further boost the catalyst applications of this material that we are going to discuss later.

### Morphological Study Based on SEM and TEM

**Figure 5:**
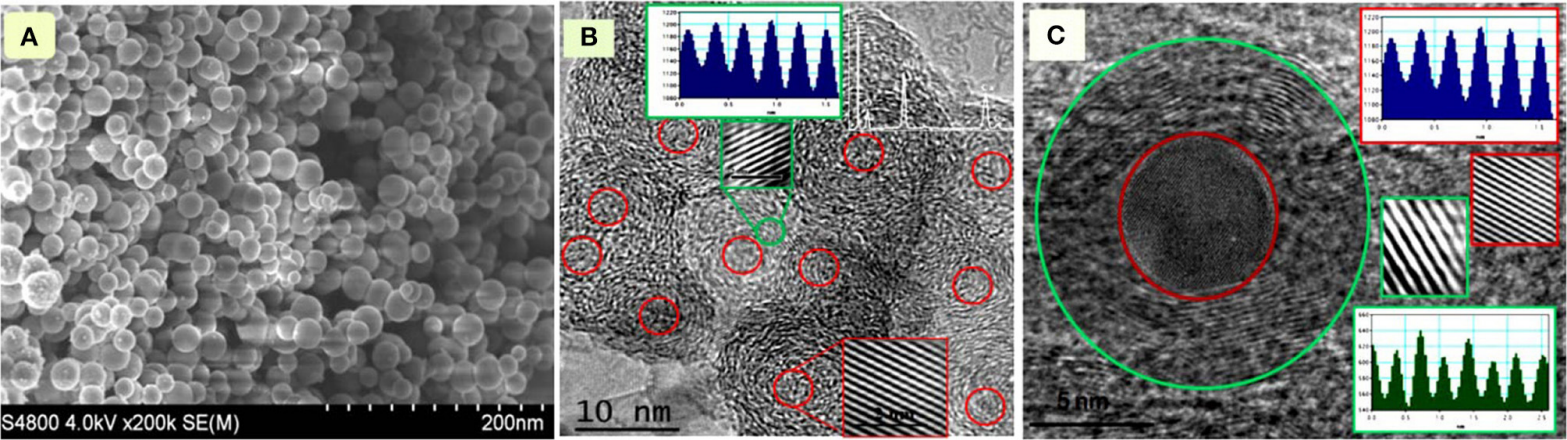
**(A)** SEM, **(B)** TEM, and **(C)** HR-TEM of the GS-CNOs synthesized at 900°C, 1 h.

For morphology analysis first of all we studied SEM of synthesized GS-CNOs, that shows all particles are well spherical and nanosize (Figure 5A). For further verification we did the TEM analysis (Figures 5B,C). The TEM characterization has been widely employed to visualize structural morphology of the nanomaterial in more details; especially here we used it to deeply study GS-CNOs structure morphology and existence of C12A7:e^−^ in synthesized material. Almost perfect GS-CNOs without a distinct amount of graphene nano-ribbons or other carbon phases were obtained at 900°C for 1 h, under applied N_2_ flow. Figures 5B,C shows that the C12A7:e^−^ particles are embedded in GS-CNOs, and are further clarified by the HR-TEM image inset Figure 5C, looks like not fully crystalline because under these applied conditions, C12A7:e^−^ become fully crystalline at >950°C (Khan et al., 2016, 2018a,b,d,e, 2020). The inset Figure 5C HR-TEM image with spacing well agree with C12A7:e^−^ (420) peak and another HR-TEM images of GS-CNOs like structure with a lattice spacing of ~0.33 nm, due to graphitization by annealing at 900°C for 1 h (Zhou et al., 2011). Now we are finally going to discuss one more important parameter of synthesized GS-CNOs for the electrocatalyst application, i.e., the conductivity measurement.

### Conductivity

**Figure 6:**
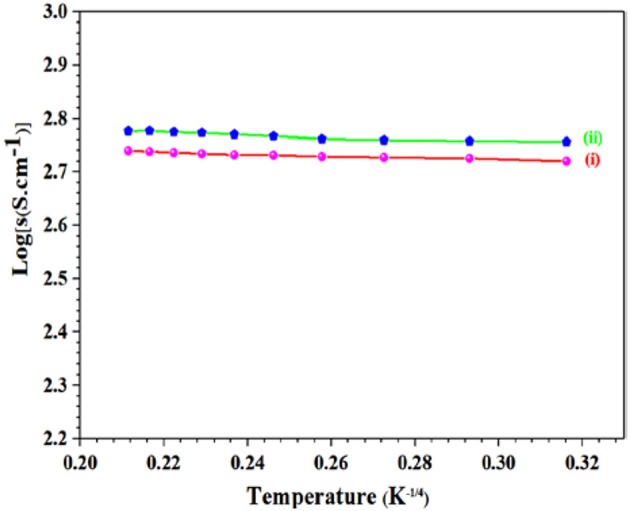
Log(σ) vs. 1/temperature [T^−1/4^(K)] graph of synthesized material, (i) Aero-gel, and (ii) GS-CNOs.

Charge transportation in electrocatalytic materials is important for laboratory research as well as regarding to large scale industrial applications. Conductivity of GS-CNOs composite, with variable temperature range from 90 to 500 K, was calculated by means of Pt-electrodes formed in a 4-prob configuration (Figure 6). Small quantity of binder was added to GS-CNOs powder and then pressed it in pellet shape at about 150 MPa and electrical contact was enhanced on pellet sample with Pt-paste. The vacuum pump was linked with chamber after sample adjustment and temperature was controlled by Liquid nitrogen and thermocouple. Conductivity of GS-CNOs and Aero-gel, synthesized samples at 900°C for 1 h was 250 and 140 S.cm^−1^, respectively. Hence first time here we proposed synthesis of such a high conductive nano-sized GS-CNOs with highest BET surface area (Borgohain et al., 2014). The ORR is a significant process in the fuel cells technology, which is highly desirable to satisfy the urgent demand of the renewable energy. Therefore, we are going to discuss application side of this high surface area GS-CNOs composite, by ORR for fuel cell technology.

## Electrocatalyst Activity and Stability of the GS-CNOs Toward ORR for Fuel Cell

**Figure 7:**
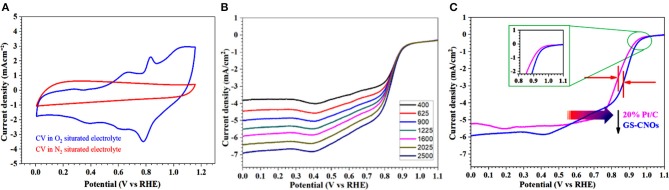
**(A)** CVs curves in O_2_ and N_2_ saturated 0.1 M KOH solution, at a scan rate of 50 mV·s^−1^. **(B)** LSV curves with various rotation rates at a scan rate of 10 mV·s^−1^. **(C)** Comparison of LSV curves of GS-CNOs (blue curve) and benchmark 20% Pt/C (pink curve) at same rotation rate of 1,600 rmp.

Electrochemical estimation of catalysts toward ORR was achieved using RDE technique. The CV calculation of the moderate conductive high surface area GS-CNOs were performed in aqueous O_2_ saturated 0.1 M KOH solution with a scan rate of 100 mV/s (Khan et al., 2018e). The GS-CNOs show a cathodic current in O_2_ saturated basic medium but not for N_2_ saturation (Figure 7A).

Now we are going to further inside the ORR results by calculating the LSV curves. The LSV curves are important to obtain the onset potential, half wave potential and current density, those plays very important role in fuel cells efficiency. Figure 7B shows the LSV curves of GS-CNOs in O_2_ saturated, 0.1 M KOH solution under rotation speeds of 400–2,500 rpm. LSV curves of GS-CNOs demonstrate that their current densities strongly rely on diffusion of oxygen with changing rotational speeds, that is normally observed (Khan et al., 2018e). Figure 7C shows comparison of LSV curves of GS-CNOs and benchmark 20% Pt/C at 1,600 rpm. The onset potential, half wave potential, and limited current density of GS-CNOs were 1.03 eV, 0.88 V, and 5.9 mA.cm^−2^, respectively, were higher than the benchmark 20% Pt/C electrode (1.02 eV, 0.82 V, and 5.2 mA.cm^−2^). This may occurs because of work-function difference between GS-CNOs and C12A7:e^−^, which causes electron transfer from low work function C12A7:e^−^ to GS-CNOs (Khan et al., 2018e). That's Hence, GS-CNOs with moderate conductivity, causes less resistance, and elevated binding energy, decreasing detrimental strongly adsorbed intermediates, thus boost the catalytic property. These facts have suitably show high intrinsic activity of GS-CNOs for ORR (Khan et al., 2018e).

**Figure 8:**
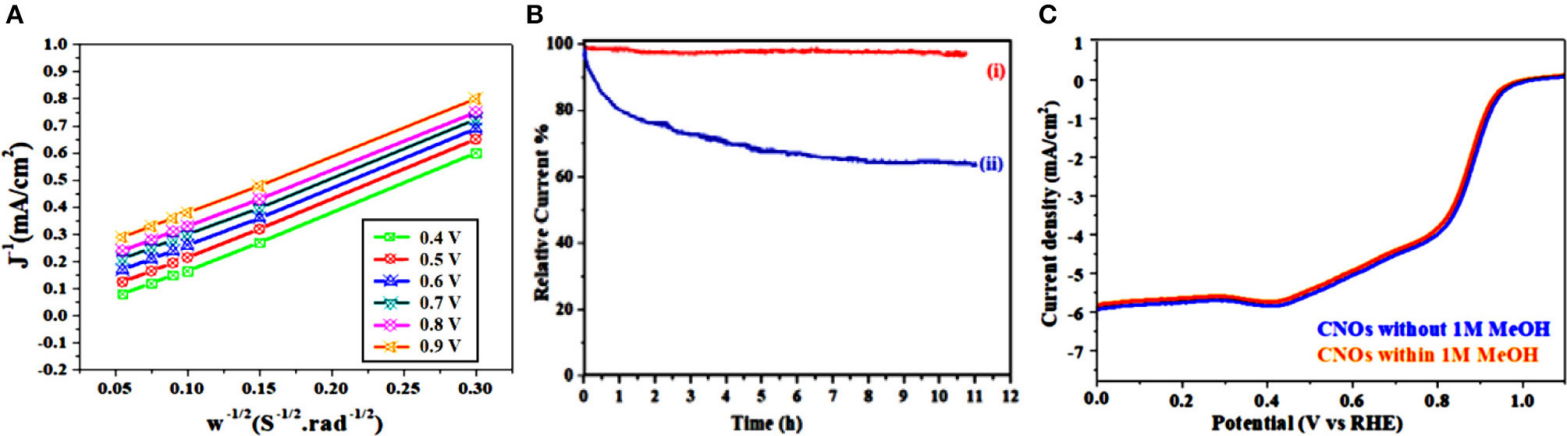
**(A)** K-L plots. **(B)** Chronoamperometric response at 0.6 V of (i) GS-CNOs and (ii) 20% Pt/C electrode. **(C)** LSV curves without (blue curve) and with (red curve) 1 M MeOH.

Finally to characterize electrons transfer (n) in GS-CNOs, by using Koutecky-Levich (K-L) plots (Figure 8A) (Khan et al., 2018e). The K-L plots with good linearity and parallelism, show the electron transfer number about 3.89–3.98 at potential 0.3–0.8 V, showed almost 4 electron systems, similarly to that of the benchmark 20% Pt/C as well as previously published reports about the GS-CNOs (Khan et al., 2018e). Figure 8B shows that for GS-CNOs almost no changes were observed (Figure 8Bi) but in contrast for state-of-the-art 20% Pt/C electrode, about 39% decrease were observed, which shows that the GS-CNOs stability is better than standard 20% Pt/C catalyst (Figure 8Bii) (Khan et al., 2018e). The kinetic losses primarily for the 20% Pt electrode maybe result from the dissolution of active metal sites due to the active carbon black corrosion but here in our this case we did not add any extra carbon black to further enhance the conductivity of GS-CNOs electrocatalyst. Therefore, relatively high degree of the graphitization is essential for Pt-free catalyst stability to enhance the corrosion resistance of GS-CNOs embedded C12A7:e^−^ with comparatively more stablity (Li et al., 2018).

**Figure 9:**
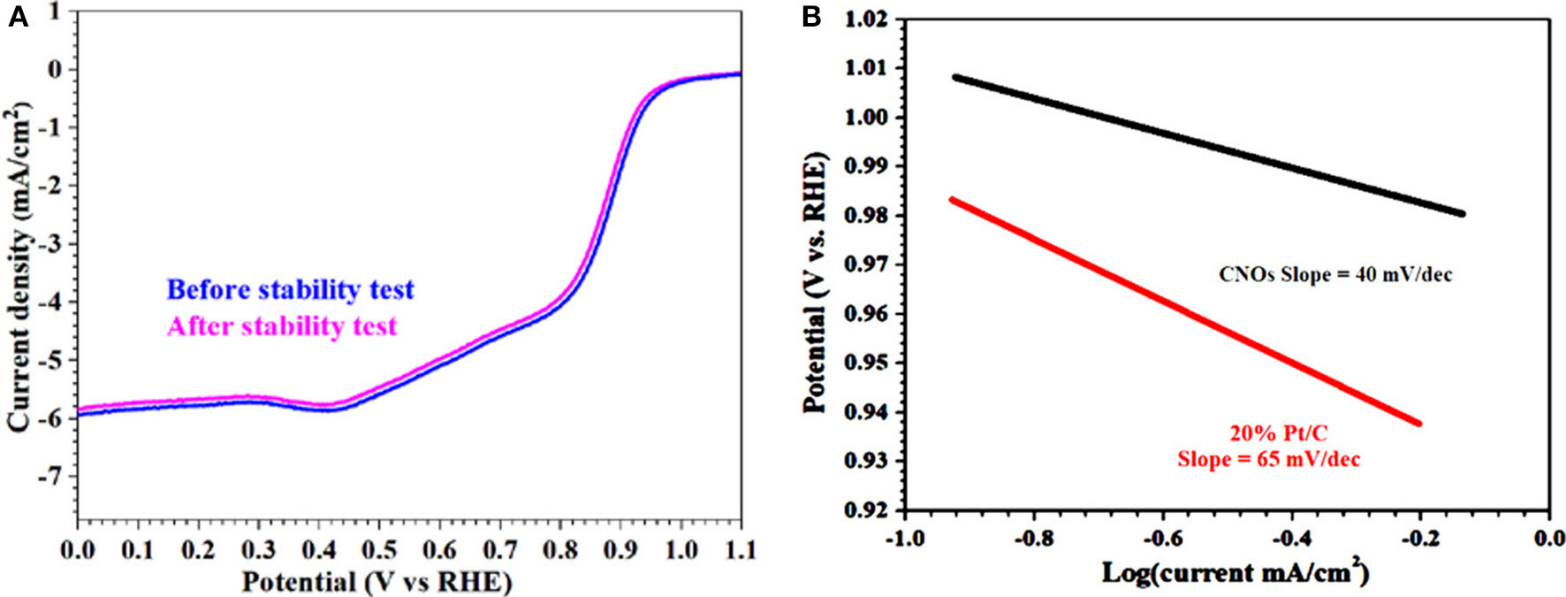
**(A)** LSV curves of GS-CNOs before and after 20,000 potential cycles in O_2_-saturated 0.1 M KOH solution and rotation speed: 1,600 rpm. **(B)** Tafel plots calculated from RDE polarization curves.

Similarly, durability and resistance to the fuel molecule (i.e., methanol) also have a potential importance especially in current industrialized alkaline fuel cell technologies. The most practical and important point is the effect of methanol crossover on the ORR activities of the GS-CNOs and was tested. It showed only about 1% loss (Figure 8C). Hence, this behavior shows that the GS-CNOs have potential application in direct methanol fuel cells. These results indicate that the GS-CNOs composite have improved long-term durability for ORR and enhanced opposition to methanol crossover as compare to the benchmark 20% Pt/C. The next important aspect regarding to the fuel cell application is the stability of the electrocatalyst. The synthesized GS-CNOs loaded on electrode were cycled from 0 to 1.2 V at 50 mV s^−1^ in O_2_-saturated 0.1 M KOH solution. The GS-CNOs also exhibits excellent stability (Figure 9A). After continuous 20,000 cycles, the half-wave potential of the GS-CNOs is almost maintained with only a shift of ≤1 mV but about ~20 mV shift has been observed for benchmark 20% Pt/C catalyst (Khan et al., 2018e). Lastly, Figure 9B shows that GS-CNOs show the Tafel slope of ≈40 mV dec^−1^, greatly lesser than benchmark 20% Pt/C (≈65 mV dec^−1^), showing it as a very active electrocatalyst for ORR.

**Figure 10:**
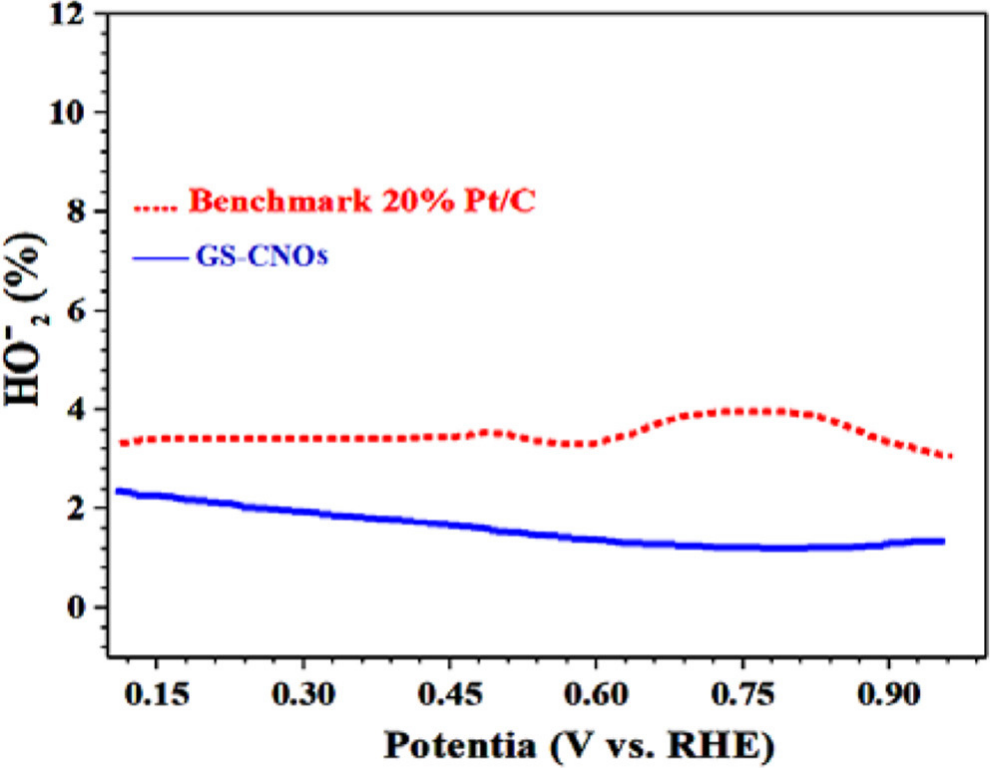
${\text{HO}}_{2}^{-}$ % yield for oxygen reduction reaction.

The high activity of the GS-CNOs is ascribed combined effect of the GS-CNOs, and C12A7:e^−^, which probably persuade favorable changes in density of state of metallic sites of catalysts. So, the synthesis method of GS-CNOs is very facile and synthesized GS-CNOs are more stable/durable, low cost and highly active as compared to that of standard 20% Pt/C standard electrode. The stability maybe because of C12A7:e^−^ embedded in GS-CNOs and can easily transfer electron to the GS-CNOs due to difference in work function and hence enhanced ORR performance (Jiang et al., 2016). The lower work function and higher electron density of C12A7:e^−^ greatly favor the electron transfer to GS-CNOs, and lead to an easier oxygen adsorption and reduction on active GS-CNOs sites, and thus an enhanced ORR activity. The ${\text{HO}}_{2}^{-}$ % yield for ORR measured by the RRDE method shows that the GS-CNOs sample has less ${\text{HO}}_{2}^{-}$ % yield as compared to benchmark 20% Pt/C (Figure 10). Similarly, electrocatalytic results of aero-gel are shown in Figures S3A,B, has less ORR activity and also less stable (Figure S3C) as compared to the GS-CNOs.

## Polarization and Power Density Curves of Alkaline Anion Exchange Membrane Fuel Cells (AAEMFC)

**Figure 11:**
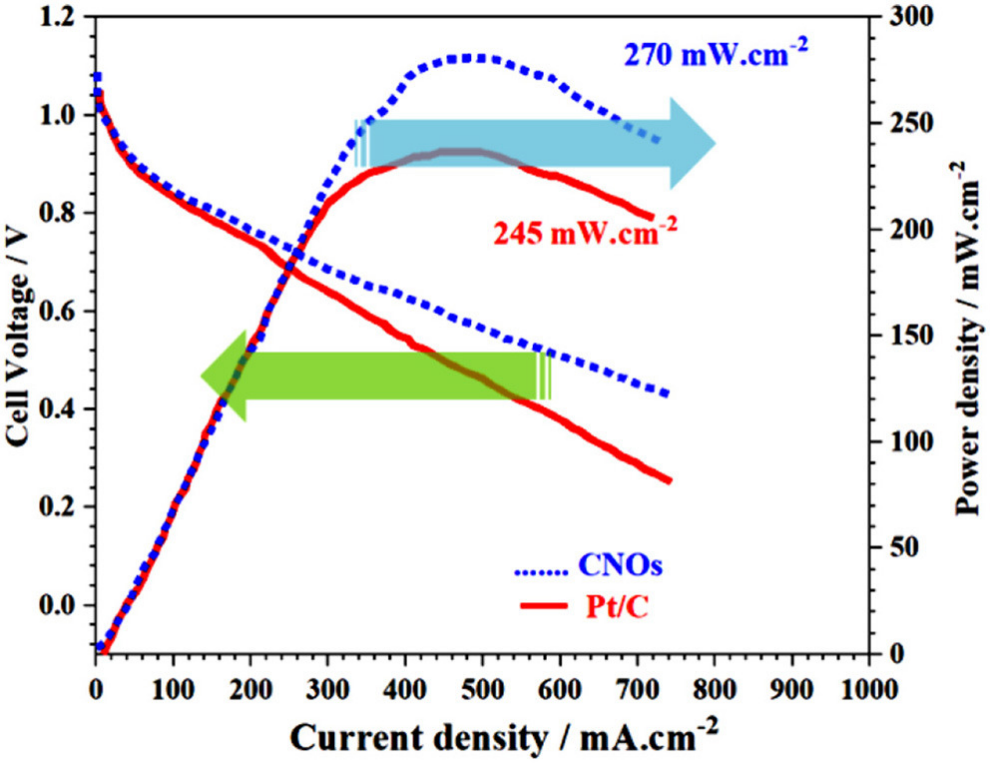
Cell voltage and power density vs. current of GS-CNOs composite catalyst as a cathode for AAEMFC. Fuel cell working conditions; catalyst loading: 0.4 mg cm^−2^, operation temperature: 60°C; H_2_ and O_2_ flow rates: 200 cc min^−1^; relative humidity of anode and cathode: 100%; and membrane: A201 (Tokuyama).

The GS-CNOs composite catalyst was used as a fuel cell cathode, and its performance in the real system was assessed by membrane electrode assembly (MEA) analysis in the AAEMFC. The polarization and power density curves of the H_2_/O_2_ AAEMFCs with GS-CNOs composite and Pt/C are shown in Figure 11.

At an operation temperature of 60°C, open circuit voltages of Pt/C and GS-CNOs composite-based AEMFCs were 1.02 and 1.03 V, respectively, which are in good agreement with trend of the onset potential in LSV. The maximum power densities can reach up to 245 and 270 mW/cm^2^ for Pt/C and GS-CNOs composite, respectively (Xin et al., 2013; Sultan et al., 2018). In short, the atomic-level detail understanding of the ORR mechanism of GS-CNOs synthesized by this new method are still in its very early stages because of the high complexity of ORR kinetics but we can conclude that first time synthesis of gram level GS-CNOs, where C12A7:e^−^ acts as a catalyst, boosted its electrochemical properties in fuel cell and can replaced traditional commercially used expensive but relatively less stable 20% Pt/C electrode.

## Conclusion

In summary, herein, a twofold GS-CNOs-C12A7:e^−^ composite activity based on the ORR electrocatalytic measurements were studied. Briefly, first we investigated controlled synthesis of the GS-CNOs based on C12A7:e^−^ catalyst. Almost spherical shape, nano-size GS-CNOs with conductivity ~250 S.cm^−1^, and highest estimated specific surface area, 214 m^2^.g^−1^, were synthesized by facile single step synthesis citrated sol-gel method. High BET surface area with well spherical shaped nanosized GS-CNOs encouraging the ORR performance with onset potential of 1.03 eV, half wave potential of 0.88 V, and limited current density of 5.9 mA.cm^−2^, higher than commercially bought standard 20% Pt/C (1.02 eV, 0.82 V, 5.2 mA.cm^−2^), with power densities of 270 mW/cm^2^. Hence, we explored large scale synthesis of the GS-CNOs composite in gram scale without further purification which provides efficient approach to prepare novel, low cost, non-Pt catalyst for future fuel cells. This work opens up a new way in the rational design of the new electrocatalysts, and reflects critical influence of the C12A7:e^−^ as a catalyst on synthesis and applications in ORR with low work function and high electron concentration as support material for GS-CNOs composite in ORR catalysis in fuel cell.

## Data Availability Statement

The raw data supporting the conclusions of this article will be made available by the authors, without undue reservation, to any qualified researcher.

## Author Contributions

KK, AT, and MA gave the idea and wrote the paper. YZ, RW, SK, QK, and MR help in writing and experimental work. HZ, ZO, and ZG provided lab facilities.

### Conflict of Interest

The authors declare that the research was conducted in the absence of any commercial or financial relationships that could be construed as a potential conflict of interest.
