# Supplementary material for: Facile Synthesis of Mayenite Electride Nanoparticles Encapsulated in Graphitic Shells Like Carbon Nano Onions: Non-noble-metal Electrocatalysts for Oxygen Reduction Reaction (ORR)
Source: Front Chem. 2020 Jan 22;7:934. doi: 10.3389/fchem.2019.00934 (PMC6987263; doi:10.3389/fchem.2019.00934)
Supplement: Supplementary file 1 [file Table_1.DOCX]

**F**acile **synthesis of** **Graphitic Shells like carbon nano onions: Non-noble-metal electrocatalysts for oxygen reduction reaction (ORR)**

Karim Khan^1,2, 4^*^,+^, Ayesha Khan Tareen^4,+^, Muhammad Aslam^3, +^, Yupeng Zhang^4^, Renheng Wang^4^, Sayed Ali Khan^5^, Qudrat Ullah Khan^5^, Muhammad Rauf ^6^, Han Zhang^4^*, Zhengbiao Ouyang^2^* and Zhongyi Guo^1,*^.

1. *Advanced electromagnetic function laboratory, Dongguan university of Technology, Dongguan, Guangdong, People republic of China.*
2. *College of Electronic Science and Technology, and THz Technical Research Center of Shenzhen University, Key Laboratory of Optoelectronics Devices and Systems of Ministry of Education and Guangdong Province Shenzhen University, Shenzhen 518060, P.R. China.*
3. *Government Degree college PaharPur, Gomel University, Dera Ismail Khan, K.P.K., Islamic Republic of Pakistan.*
4. *Shenzhen Engineering Laboratory of Phosphorene and Optoelectronics, and SZU‐NUS Collaborative Innovation Center for Optoelectronic Science and Technology, Shenzhen University, Shenzhen, 518060, China.*
5. *Shenzhen Key Laboratory of Flexible Memory Materials and Device, Collage of Electronic Science and Technology, Shenzhen University, Nanhai Ave. 3688, Shenzhen, Guangdong 518060, P.R. China.*
6. *College of Chemistry and Environmental Engineering, Shenzhen University, Shenzhen, Guangdong 518060, PR China.*

****Corresponding author:*** ***Karim Khan*:***karim_khan_niazi@yahoo.com,* **Zhang Han***:* [*hzhang@szu.edu.cn*](mailto:hzhang@szu.edu.cn)*,* and ***Zhengbiao Ouyang****:* [*zbouyang@szu.edu.cn*](mailto:zbouyang@szu.edu.cn)*.*

*.*

**Electrocatalytic data calculations and conversion**

All the obtained potential data versus SCE in this study were converted to potential versus RHE using the following formula;

$E\left( vs.RHE \right)= E\left( vs. SCE \right)+ E_{SCE}^{^{\circ}}+0.0591 pH (1)$

The number of electrons transferred and hydrogen peroxide production were evaluated from RRDE measurement. The number of electron transfer (n) during ORR testing is,

$n=4\times\left( \frac{I_{d}}{{\frac{I_{r}}{N}+I}_{d}} \right) (2)$

Where, I_d_ and I_r_ are disk and ring current densities, respectively, and *n* is the current collection efficiency of the Pt ring which is 0.37. The percentage of peroxide (% HO_2_^−^) during the ORR process is

$$\%H_{2}O^{-}=200\times\left( \frac{\frac{I_{r}}{N}}{{\frac{I_{r}}{N}+I}_{d}} \right) (3)$$

The electron transfer number and kinetics of ORR from RRD measurement were evaluated using the following Koutecky-Levich equation;

$$\frac{1}{j}= \frac{1}{j_{l}}+ \frac{1}{j_{k}}= \frac{1}{{B\omega}^{0.5}}+\frac{1}{j_{k}} (4)$$

$$B=0.64nFAC_{0}D_{0}^{2/3}V^{-1/6} (5)$$

$$J_{k}=nFkC_{0} (6)$$

Where *j* is the measured current density, *j*_l_ and *j*_k_ are the limiting and kinetic current densities, *B* is the slope of K‐L plots, *ω* is the rotation rate of the disk electrode, *n* is the electron transferred number in ORR, *F* is the Faraday constant (96,485 C mol^-1^), *A* is the geometric area of electrode (A = ~0.196 cm^-2^), *C*_0_ is the concentration of O_2_ which is 1.2×10^-6^ mol cm^-3^ in 0.1 m KOH, *D*_0_ is the O_2_ diffusion coefficient (1.9 × 10^-5^ cm^2^ s^-1^), *V* is the kinematic viscosity of the solution (0.01 cm^2^ s^−1^), and *k* is the electron‐transfer rate constant.

**Fuel Cell Test**

The catalyst was used as a fuel cell cathode, and its performance in the real system was assessed by membrane electrode assembly (MEA) analysis in the AAEMFC. For the preparation of the cathode, the catalyst and commercial ionomer (50 wt%) were well dispersed in isopropyl alcohol (IPA) via sonication. Subsequently, the homogeneous ink of catalyst (0.1 mg cm^-2^) as a cathode and Pt/C (0.1 mg cm^−2^, which contain loaded quantity of Pt is ~20 μg cm^- 2^) as an anode were loaded on the active area of the gas diffusion layer (GDL). The MEA was fabricated by sandwiching the potassium hydroxide-doped Tokuyama membrane between the cathode and anode. Finally, the MEA was conducted in a single cell mode, which comprises serpentine flow field channels in the graphite plates. The steady‐state polarization experiment (cell voltage and power) of the assembled MEA was measured at 60 °C by keeping a humidified flow (100% relative humidity) of hydrogen and oxygen at the flow rate of 200 cc min^-1^.


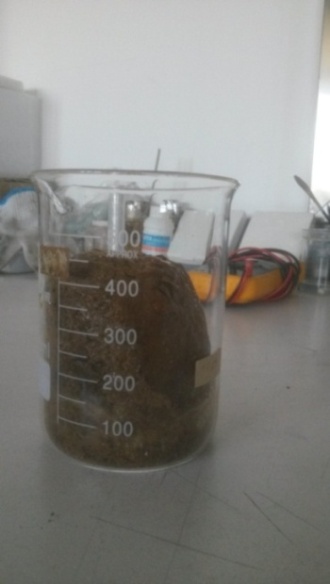

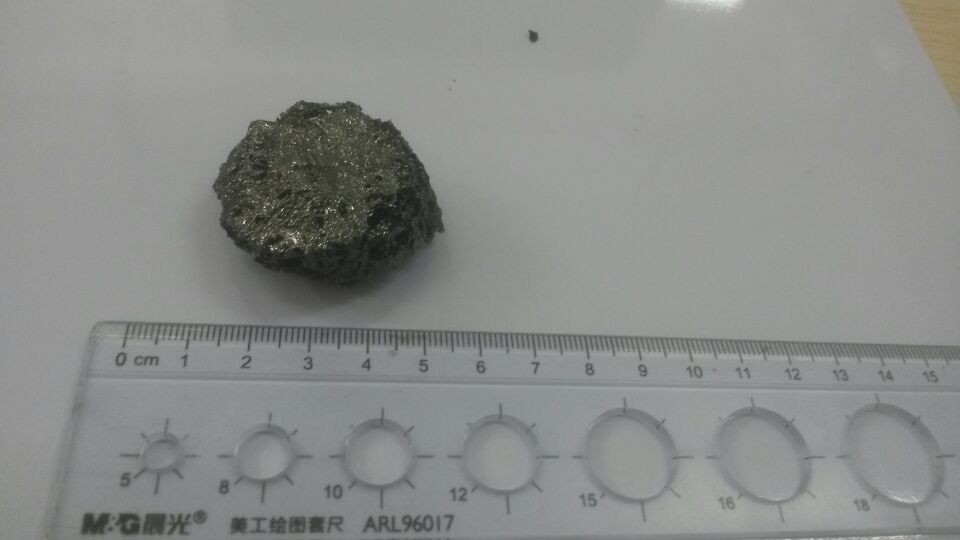




(a) (b) (c)

**Fig.S1. (a)** Precursors dried-gel (290 °C, 2h), **(b)** Optical image of Aero-gel (900 °C, 1h) and **(c)** SEM of Aero-gel (900 °C, 1h).


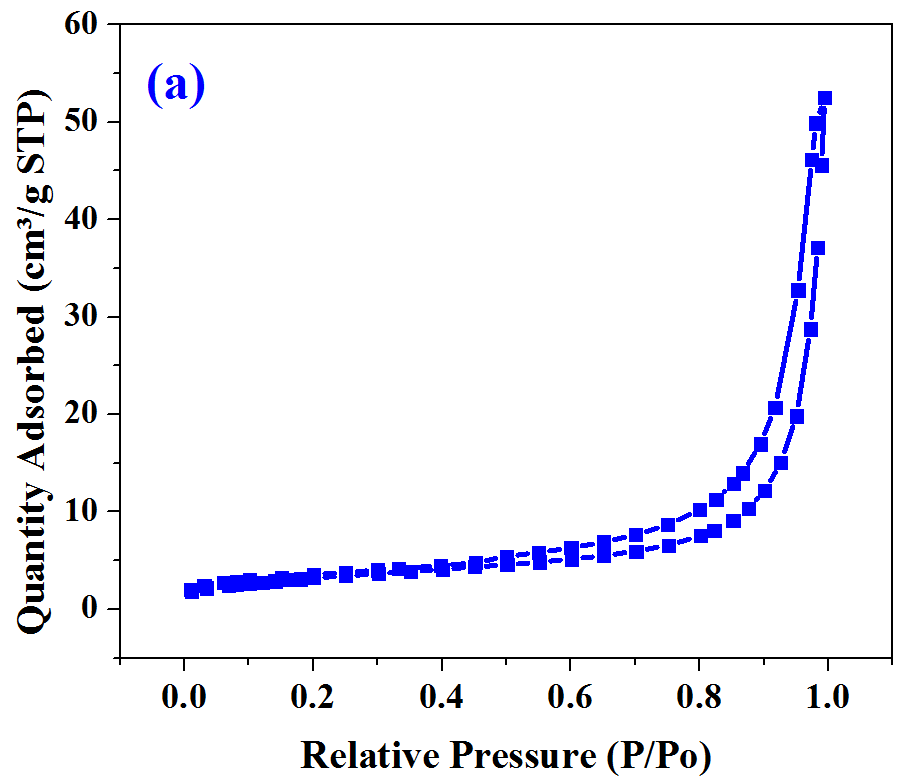

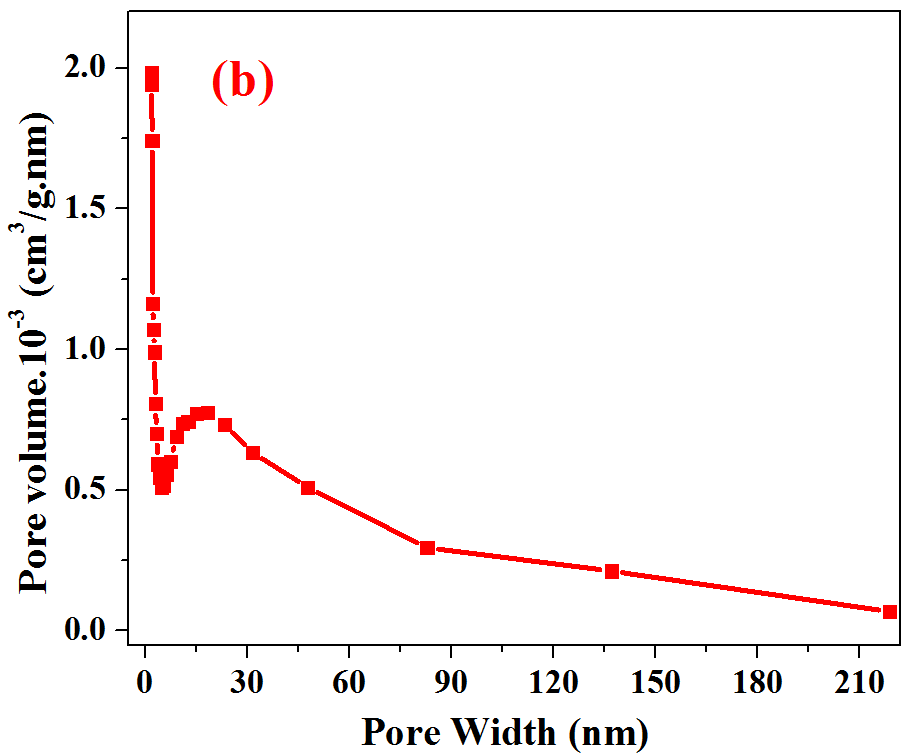


**Fig.S2.** BET of aero-gel synthesized at 900 °C, 1h.


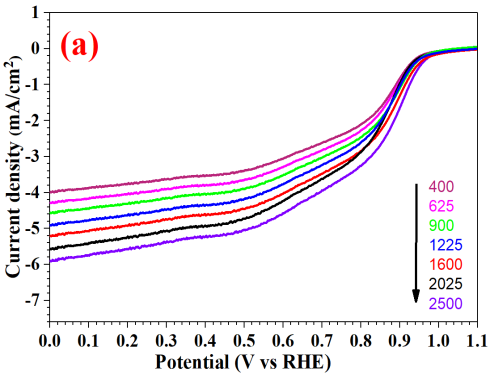

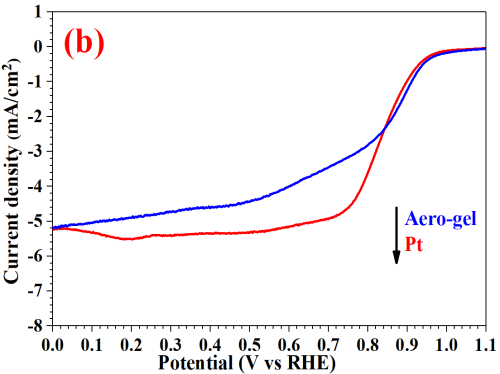

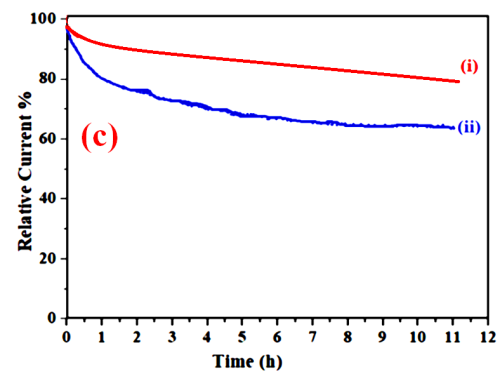


**Fig.S3. (a)** LSV curves with various rotation rates at a scan rate of 10 mV·s^-1^, and **(b)** comparison of LSV curves of Aero-gel (blue curve) and 20% Pt/C (red curve) at same rotation rate of 1600 rmp, **(c)** Chronoamperometric response at 0.6V of **(i)** Aero-gel and **(ii)** 20% Pt/C electrode.
